# Radiomics and machine learning for differentiating pediatric intramedullary spinal tumors: a pilot study

**DOI:** 10.1186/s41747-026-00810-2

**Published:** 2026-09-21

**Authors:** Catalin George Iacoban, Shayan Alvansazyazdi, Costanza Parodi, Behnoud Shafiezadeh Kenari, Antonia Ramaglia, Claudia Mercuri, Gabriele Gaggero, Gianluca Piatelli, Claudia Milanaccio, Davide Cangelosi, Andrea Rossi

**Affiliations:** 1 Neuroradiology Unit, IRCCS Istituto Giannina Gaslini, Genoa, Italy; 2 Clinical Bioinformatics Unit, IRCCS Istituto Giannina Gaslini, Genoa, Italy; 3 Neuro-Oncology Unit, IRCCS Istituto Giannina Gaslini, Genoa, Italy; 4 Pathology Unit, IRCCS Istituto Giannina Gaslini, Genoa, Italy; 5 Neurosurgery Unit, IRCCS Istituto Giannina Gaslini, Genoa, Italy; 6 Department of Health Sciences (DISSAL), University of Genoa, Genoa, Italy

**Keywords:** Machine learning, Nested leave-one-out cross-validation, Radiomics, Pediatric intramedullary spinal tumors

## Abstract

**Objectives:**

Conventional magnetic resonance imaging faces limitations in differentiating among pediatric intramedullary spinal tumors (IMSTs). We evaluated radiomics-based machine learning (ML) models using sagittal T2-weighted images to differentiate pilocytic astrocytomas (PA) and ependymomas (EP), independently, from other IMSTs.

**Materials and methods:**

This single-center retrospective study included pediatric patients diagnosed with IMST from 2000 to 2023. Manual tumor segmentation was performed on sagittal T2-WI, in correlation with sagittal contrast-enhanced T1-weighted images using ITK-SNAP. Radiomic features were extracted using PyRadiomics. Effect size validation assessed feature analytical suitability prior to ML implementation. Three ML models with two feature selection methodologies were deployed using nested leave-one-out cross-validation. Balanced accuracy, Matthews correlation coefficient (MCC), sensitivity, specificity, positive/negative predictive values (PPV, NPV), F1 score, area under the receiver operating characteristic curve (AUC), Youden’s *J*, and Cohen’s *d* were calculated.

**Results:**

Forty patients, aged 9.2 ± 5.4 years (mean ± standard deviation), 21 females, were analyzed. Tumors comprised PAs (*n* = 16), EPs (*n* = 11), gangliogliomas (*n* = 6), and others (*n* = 7). Cohen’s *d* was 0.356 for PAs and 0.741 for EPs. PA radiomics showed inadequate capacity, resulting in suboptimal ML performance. Random forest with least absolute shrinkage and selection operator (LASSO) achieved superior EP classification using an average of four features (Youden’s *J* = 0.243): balanced accuracy 0.834 (95% confidence interval: 0.723–0.955, *p* < 0.0001), AUC 0.838, sensitivity 0.909, specificity 0.759, PPV 0.588, NPV 0.957, and MCC 0.603.

**Conclusion:**

Radiomics-based ML differentiated EPs but not PAs from other pediatric IMSTs using sagittal T2-weighted images.

**Key Points:**

***Question*** Can radiomic-based machine learning models differentiate pediatric intramedullary spinal tumors?

***Findings*** Random forest with LASSO feature selection achieved acceptable performance in distinguishing ependymomas from other IMSTs, with balanced accuracy of 0.834, AUC of 0.838, and MCC of 0.603.

***Relevance statement*** This review highlights the emerging role of time-resolved (“4D”) approaches in postmortem imaging, demonstrating how integrating temporal information can enhance reconstruction of injury mechanisms, postmortem processes, and medicolegal interpretation, thereby expanding forensic radiology beyond static documentation toward process-oriented forensic investigation.

**Graphical Abstract:**

**Figure:**
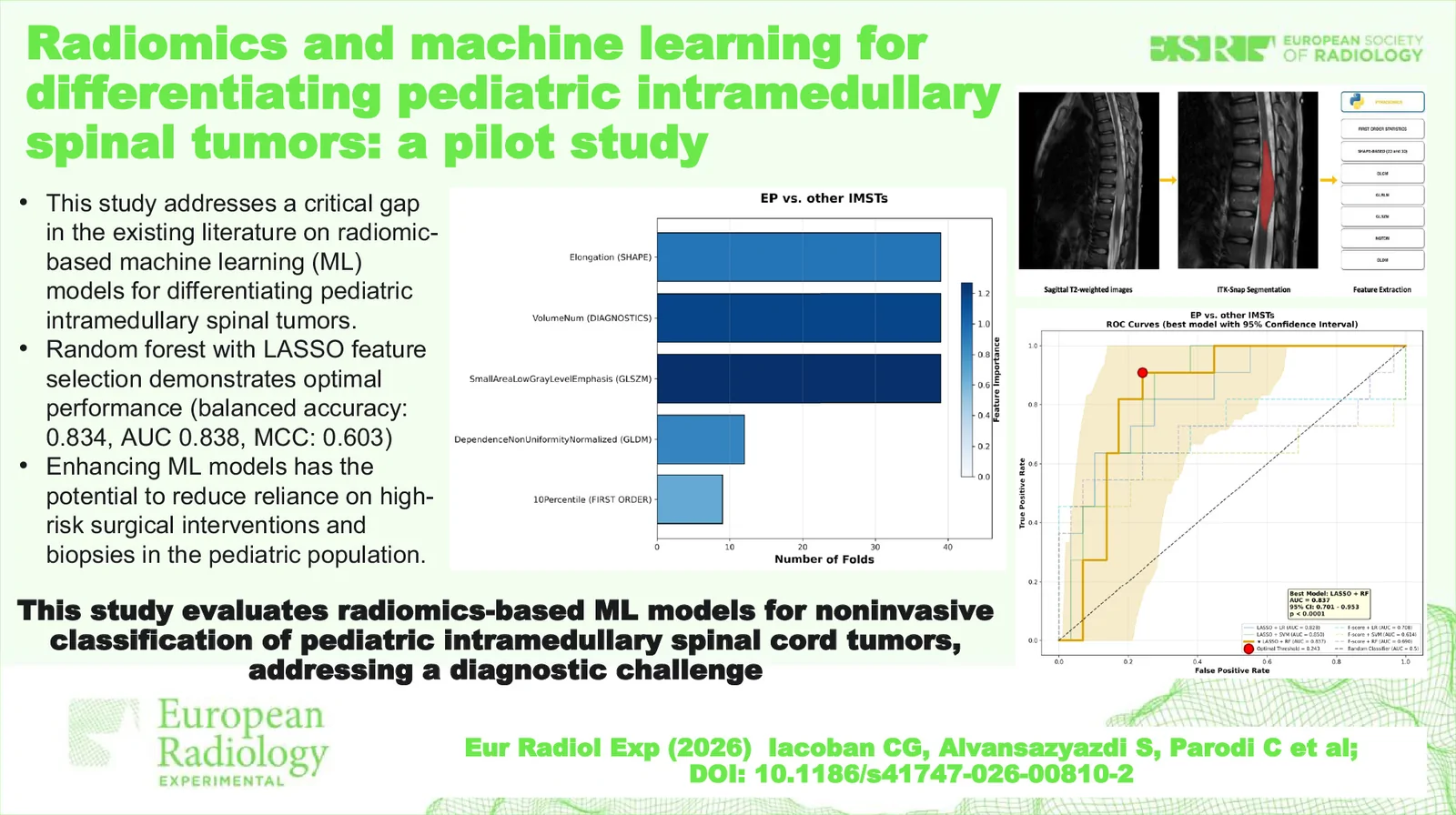

## Background

In the pediatric age group, spinal cord and cauda equina tumors accounted for 5.2% of all central nervous system tumors between 2016 and 2020, as reported by CBTRUS (Central Brain Tumor Registry of the United States), the leading histopathology-specific repository globally. Among these lesions, approximately 70–80% originate from the spinal cord [1]. Despite their infrequency, these tumors are characterized by an unfavorable clinical trajectory and entail significant perioperative risks for neurologic deficits [2].

Differentiating among pediatric spinal tumors exclusively *via* conventional magnetic resonance imaging (MRI) is challenging due to overlapping features. The differentiation is, however, crucial due to their distinct natures, therapeutic approaches, and prognostic implications. Given the inherent risks associated with biopsy, the utilization of artificial intelligence to noninvasively predict histologic tumor types emerges as a promising avenue to mitigate these risks. The past decade has witnessed the exponential development of radiomics, which involves extracting high-dimensional, mineable data from medical images, data typically imperceptible to the human eye. This information enhances diagnostic predictability both in the initial and post-therapeutic settings [3].

Radiomics, when integrated into machine learning (ML) and deep learning (DL) frameworks, has garnered significant interest as a diagnostic adjunct in spinal pathologies. Research has explored its applications across lesion classification, tumor segmentation, grading, mutation prediction, and prognosis. Noteworthy contributions include efforts to achieve fully automated tumor segmentation of astrocytomas, ependymomas, and hemangioblastomas using deep learning (DL) algorithms [4]. DL frameworks have been employed to segment and classify both tumoral (*e.g*., astrocytoma, ependymoma) and non-tumoral spinal pathologies (*e.g*., multiple sclerosis, neuromyelitis optica spectrum disorder), potentially surpassing radiologist diagnostic performance [5]. Similar work has focused on differentiating intramedullary spinal tumors (IMSTs) from tumefactive demyelinating lesions, yielding comparable results [6]. Radiomics has also been applied to tumor grading and mutation prediction. ML models leveraging neural networks and multimodal imaging techniques have successfully predicted intramedullary glioma grade, ATRX and p53 mutation status [7]. In addition, radiogenomic approaches have investigated the prediction of H3K27M mutation status in diffuse midline spinal cord gliomas [8, 9]. The application of radiomics in predicting patient prognosis has also been explored. Automated DL pipelines have been employed to segment spinal cord astrocytomas and stratify overall survival based on preoperative MR images [10].

The potential of radiomics for differentiating pediatric IMSTs remains insufficiently explored. This study seeks to address this gap by analyzing T2-weighted images (T2-WI) radiomic features of ependymomas (EP) or pilocytic astrocytomas (PA) in comparison to those of other IMSTs for ML-driven histology prediction.

## Materials and methods

This single-center retrospective observational study was approved by the Regional Ethical Committee (CER Liguria: 345/2020-DB ID 10589) on 05/10/2020.

### Patient characteristics and eligibility criteria

**Fig. 1:**
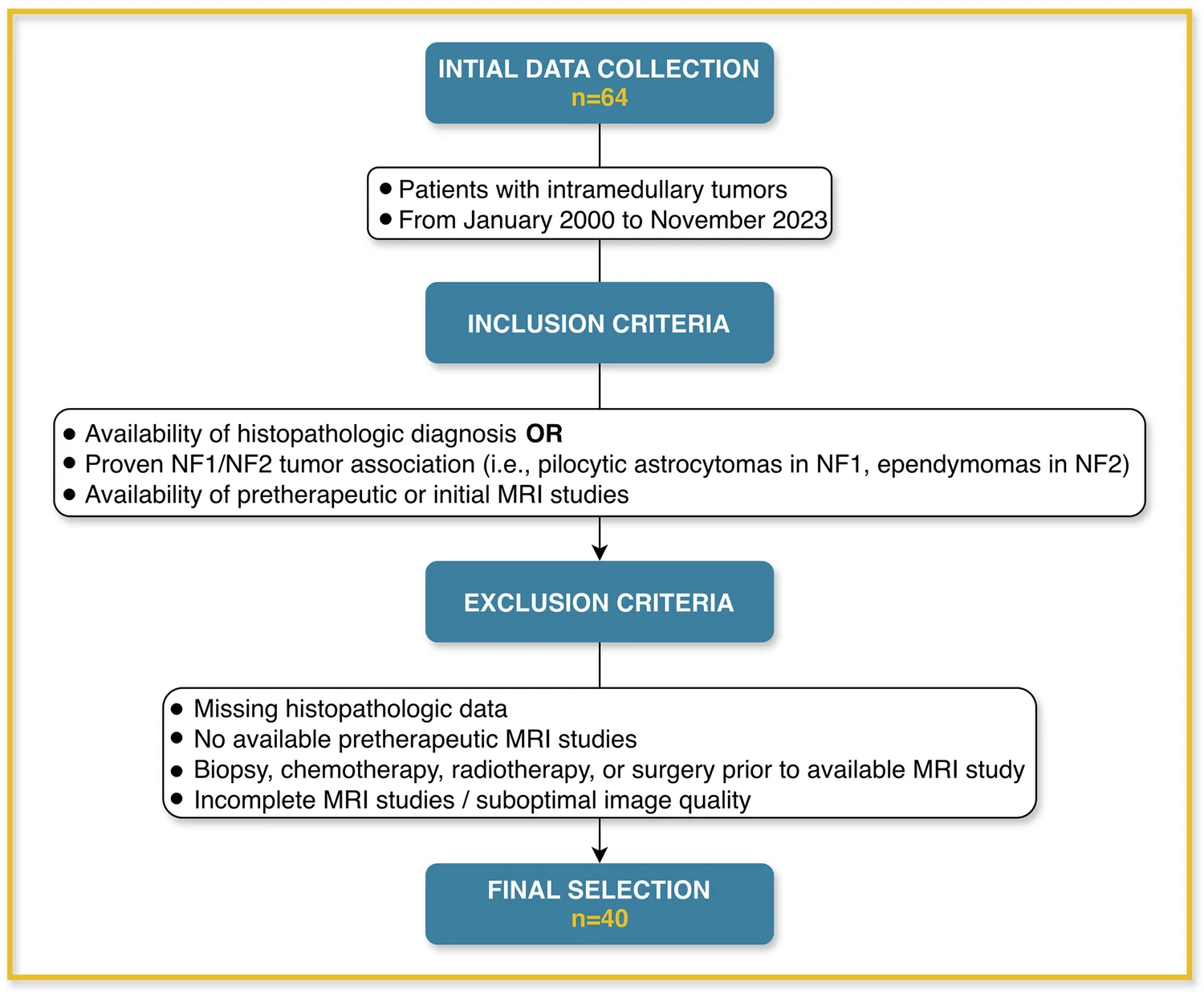
Flow diagram illustrating patient selection. NF1, Neurofibromatosis type 1; NF2, Neurofibromatosis type 2

A retrospective search for IMSTs was undertaken within the local patient database encompassing admissions to our institution from 2000 to 2023. Inclusion criteria included patients possessing either histopathological confirmation or presumed diagnosis *via* established associations with phacomatoses (specifically, pilocytic astrocytomas in neurofibromatosis type 1 and ependymomas in neurofibromatosis type 2). Additionally, eligible cases necessitated the availability of initial diagnostic or pretherapeutic imaging studies, inclusive of MRI protocols featuring sagittal T2-WI and post-contrast T1-weighted images (T1-WI). Exclusion criteria entailed unavailability of initial or pretherapeutic MRI scans, incomplete MRI studies, poor image quality, a history of biopsy, surgery, radiotherapy, and/or chemotherapy before available imaging, or inaccessible histopathologic exams (Fig. 1).

### Image acquisition

Images were obtained using either the in-house available MRI scanners (1.5-T Intera Achieva and 3-T Ingenia D-Stream, Philips Healthcare, Best, Netherlands) (*n* = 36) or external MRI scanners in cases where initial investigation was performed elsewhere (*n* = 4; 1.5-T). All included studies consisted of an imaging protocol that included sagittal turbo spin-echo T2-WI and sagittal turbo spin-echo T1-WI acquired before (available only in 29 cases) and following intravenous gadolinium-based contrast injection (gadoteric acid; Dotarem®, Guerbet, France). The protocol default parameters ranged between the following values. For T2-WI: in plane resolution = 0.30 × 0.30 – 0.60 – 0.60 mm^2^; slice thickness = 3–4 mm, repetition time = 2,110–4,675 ms, echo time = 84–150; and flip angle = 90–160°. For pre- and post-contrast T1-WI: in plane resolution = 0.30 × 0.30 – 0.60 – 0.60 mm^2^; slice thickness = 3–4 mm, repetition time = 360–762 ms; echo time = 6–25 ms, flip angle = 70–160°.

### Radiomic analysis

The images were anonymized using the Horos software (v. 3.3.5, OsiriX™, The Horos Project, United States) by removing direct personal identifiers from the Digital Imaging and Communications in Medicine (DICOM) metadata. Prior to segmentation, a detailed assessment was conducted to precisely identify tumor location, length, the extent of edema, cystic or necrotic features, contrast enhancement patterns, and the presence of syringohydromyelia. Purely manual segmentations on sagittal T2-WI were performed by a junior radiologist possessing 5 years of experience in neuroradiology (C.G.I.). Due to the heterogeneity of tumor phenotypes, with some tumors consisting entirely of solid enhancing, solid non-enhancing, or cystic/necrotic components, the entire tumor was segmented as a single block. Post-contrast T1-WI were utilized to achieve more accurate segmentation. Segmentation was restricted to the tumor core, defined by visible lesion margins and contrast enhancement, when present, with deliberate exclusion of peritumoral hyperintensity on T2-WI, which may represent a heterogeneous mix of edema, reactive change, and microscopic infiltration that cannot be reliably distinguished on conventional MRI and could bias radiomic features. A pediatric neuroradiologist (A.Ra., 5 years’ experience) subsequently reviewed and validated the initial segmentations, adjusting where necessary. Any discrepancies were resolved by consensus following a detailed re-evaluation of the images. Both reviewers remained blinded to all clinical and histopathological information.

Segmentation was performed using ITK-SNAP software (v. 3.8.0), whereas radiomic feature extraction solely out of T2-WI was carried out using the PyRadiomics package (v. 3.0.1), an open-source software available at https://github.com/Radiomics/pyradiomics.

**Fig. 2:**
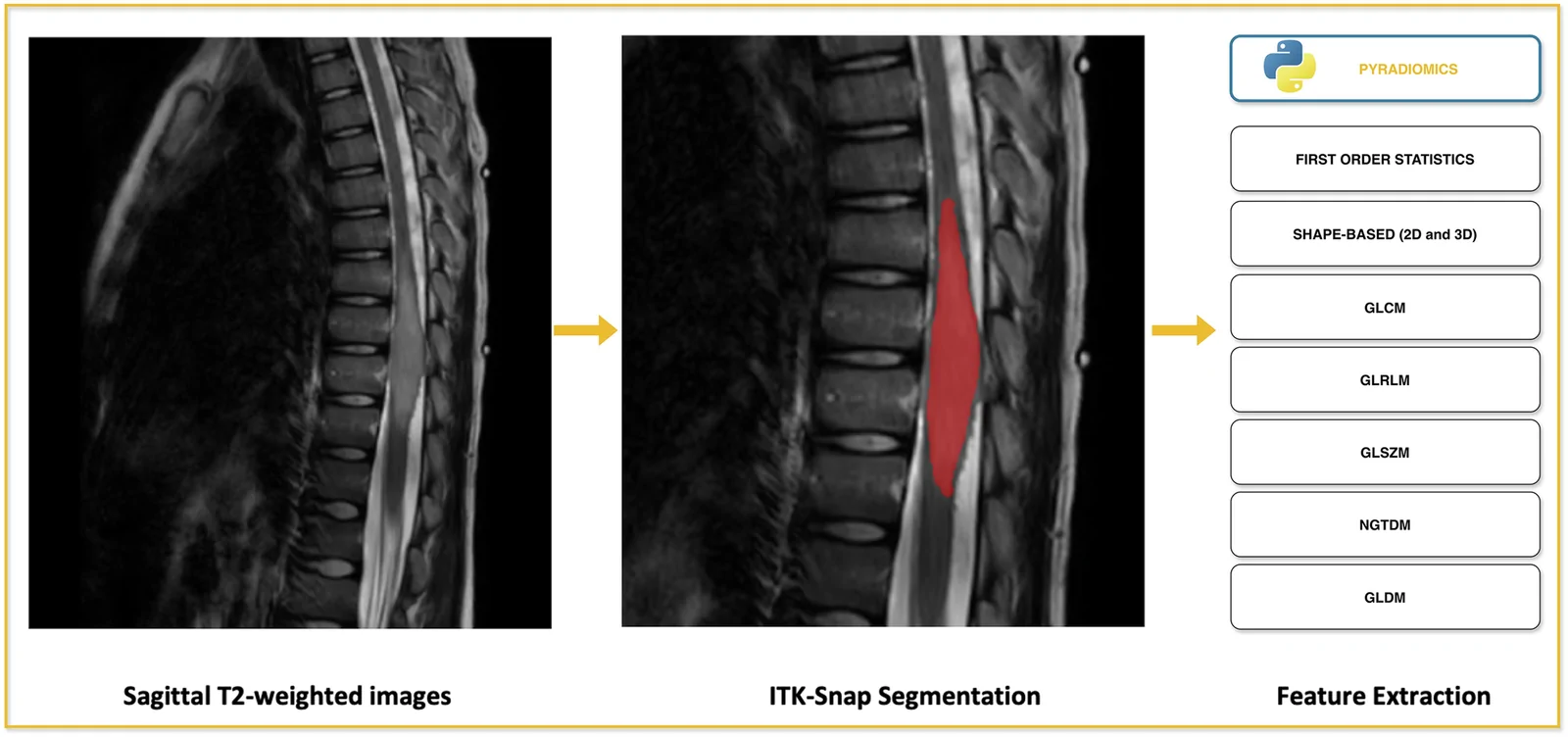
Radiomic workflow. (1) T2-weighted imaging acquisition of the spine and neck area in the sagittal plane. (2) Manual segmentation of the intramedullary lesions using ITK-SNAP software (default mode: “paintbrush”). (3) Computation of 120 radiomic features *via* the PyRadiomics package. GLCM, Gray level co-occurrence matrix; GLRLM, Gray level run length matrix; GLSZM, Gray level size zone matrix; NGTDM, Neighboring gray tone difference matrix; GLDM, Gray level dependence matrix

PyRadiomics allows for the extraction of features adhering to the definitions set by the Image Biomarker Standardization Initiative [11]. The PyRadiomics configuration statement included: (1) Z-score image normalization; (2) scaling by a factor of 100; (3) a 300-voxel array shift; (4) region-of-interest mask resampling (3 × 3 × 3 mm^3^); (5) a binwidth of 25; (6) linear interpolation for resampling images; and (7) nearest neighbor for the mask [12, 13]. In accordance with recommendations outlined in the PyRadiomics documentation, we opted for a fixed binwidth [14]. The default 120 extracted features spanned various classes, including: first order statistics (19 features), Shape-based (3D) (16 features), Shape-based (2D) (10 features), gray level co-occurrence matrix (GLCM, 24 features), gray level run length matrix (GLRLM, 16 features), gray level size zone matrix (GLSZM, 16 features), neighboring gray tone difference matrix (NGTDM, 5 features), and gray level dependence matrix (GLDM, 14 features). The entire workflow is summarized in Fig. 2.

### Machine learning and statistical analysis

Prior to designing and implementing the ML workflow, we computed the effect size to understand the reliability and feasibility of using ML with our dataset and sample size. Based on previous work by Rajput et al [15], a Cohen’s *d* absolute average (|*d*|) of ≥ 0.5 reflects a sufficient discriminability outcome to support further analysis. The effect size was calculated for both EP and PA. Three classifiers, including logistic regression [16], random forest [17], and linear support vector machine (SVM) [18], using scikit-learn (v. 1.7.2) [19] and Python (v. 3.10.19) [20], were evaluated for their performance in distinguishing EP and PA from other IMSTs. Default hyperparameters were selected for each classifier: logistic regression (C = 1.0, penalty = ‘l2’, solver = ‘lbfgs’) [21], SVM (C = 1.0, kernel = ‘linear’) [22], and random forest (*n*_estimators = 100, max_depTh = 5, min_samples_split = 5) [17]. All classifiers used class_weight = ‘balanced’ to account for class imbalance. No further hyperparameter optimization was conducted; the primary objective was robust classification and validation of feature selection rather than exhaustive performance maximization.

A nested leave-one-out cross-validation (LOOCV) approach was used for the construction of each ML model. This approach allows to tackle biased and over-optimistic performance, often described in simple cross-validation [23]. The use of the full dataset allows to improve both accuracy and generalization [24, 25], as well as to account for overfitting caused by the small sample size. Moreover, due to the low number of patients, no overall hold-out division of the dataset (training/test) was employed.

Briefly, the structure of the nested LOOCV comprised a 40-fold outer loop for testing and a 39-fold inner loop for validation and number selection of retained features (k). Specifically, two feature selection strategies were evaluated within the modeling pipeline: least absolute shrinkage and selection operator (LASSO) (L1-regularized logistic regression with C = 1.0, solver = ‘saga’, max_iter = 5,000) and F-score (ANOVA F-value). For each classifier, k features were tuned from 3 to 10 *via* the inner loop, maximizing balanced accuracy [26]. The final model was trained on all inner-loop data using the optimal k and applied to the held-out patient.

All features were Z-score normalized, using *standardscaler*, within each outer fold, with the mean and standard deviation calculated solely from the training partition to prevent data leakage.

The performance of the ML models was assessed on the pooled LOOCV predictions (one out-of-sample prediction per patient). Metrics included balanced accuracy [27], Matthews correlation coefficient (MCC) [28], sensitivity, specificity, positive predictive value (PPV), negative predictive value (NPV), F1 score, and area under the receiver operating characteristic curve (AUC) [29]. AUC, 95% confidence interval (CI), and *p*-value [30] were computed through a 1,000 bootstrap method using sklearn.metrics.roc_auc_score. The two-tailed *z*-test was used to estimate the *p*-value, which tests whether the provided AUC is significantly different from 0.5 (chance classifier). Receiving operating curves were used to visualize model predictions. In addition to the conventional decision threshold of 0.5, Youden’s *J* statistic (sensitivity + specificity - 1) was applied to identify the optimal threshold that maximizes the sum of sensitivity and specificity [31]. All metrics were recomputed at this optimized threshold. Feature stability was assessed by aggregating the selection frequency of each radiomic feature across the 40 outer folds. For each model, features were ranked by how often they were retained by the feature selector after inner-loop optimization of *k*. For the best model, the mean absolute coefficient magnitude across folds was used to indicate the relative contribution of each selected feature, with larger values indicating greater importance.

Univariate statistical analyses were performed using SPSS Statistics software (v21, IBM, Armonk, NY, USA). Categorical variables were expressed as absolute frequencies and percentages. Numerical variables were reported as mean and standard deviation. Additionally, to investigate scanner-induced bias, a Fisher–Freeman–Halton’s exact test was carried out. The null hypothesis was accepted for both EP and PA (*p*-value 0.40 and 0.21, respectively) [32].

## Results

### Patient demographics

**Fig. 3:**
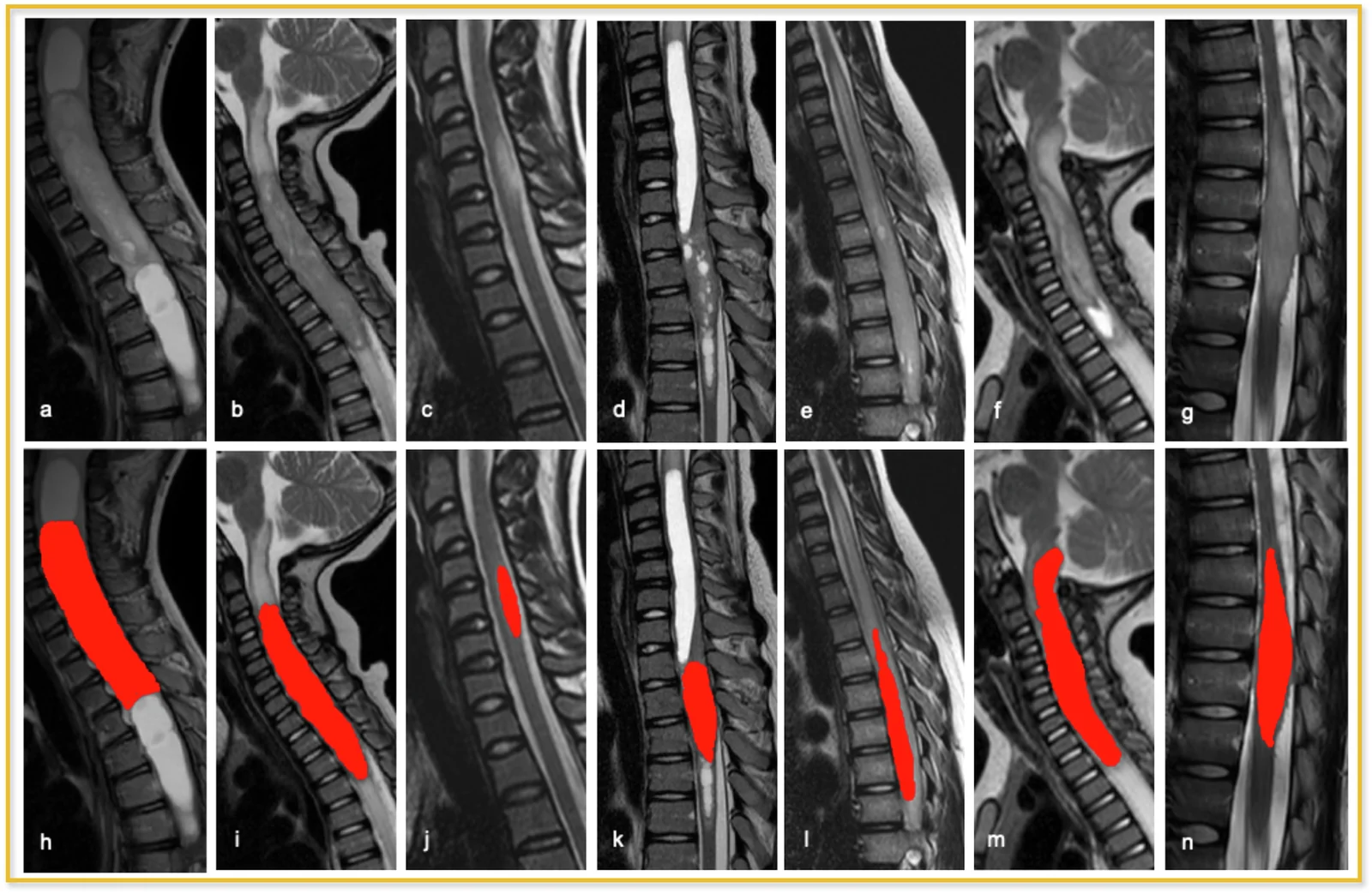
Conventional MRI features of selected IMSTs prevalent in pediatric patients. The figure includes sagittal T2-weighted images (**a**–**g**) next to manual segmentations performed using ITK-Snap (**h**–**n**). Specifically, the tumors featured are pilocytic astrocytoma (WHO Grade I) (**a**, **h**), ganglioglioma (WHO Grade I) (**b**, **i**), subependymoma (WHO Grade I) (**c**, **j**), ependymoma (WHO Grade II) (**d**, **k**), anaplastic astrocytoma (WHO Grade III) (**e**, **l**), glioblastoma (WHO Grade IV) (**f**, **m**), and diffuse midline glioma (WHO Grade IV) (**g**, **n**). Note the partially overlapping characteristics of these tumors across conventional MRI sequences. IMSTs, Intramedullary spinal tumors; MRI, Magnetic resonance imaging; WHO, World Health Organization

Based on inclusion criteria, an initial cohort of 64 patients was identified; of these, 24 were excluded (5 lacked histopathologic data, 17 had undergone prior therapy and had missing baseline studies, and 2 had incomplete MRI protocols), yielding a refined cohort of 40 eligible patients (19 males, age at scan 9.2 ± 5.4, mean ± standard deviation) (Table 1). Patients harbored a wide range of IMSTs, including 16 low-grade astrocytomas (14 PA and 2 NF1-associated low-grade gliomas), 1 subependymoma, 11 ependymomas (2 histologically confirmed and 9 NF2-associated), 6 gangliogliomas, 1 diffuse midline glioma, 3 anaplastic astrocytomas, and 2 glioblastomas (Table 1). Genetic testing, when available, revealed: 7 cases (involving PA and EP) tested negative for the BRAF mutation, 1 PA was BRAF positive, and 1 case exhibited the KIAA1549/BRAF fusion. Notably, the solitary diffuse midline glioma exhibited positivity for the H3.3K27 mutation. To extract conventional MR features, we first annotated sagittal T2-WI as shown in Fig. 3.

**Table 1:** Patient characteristics

	Mean ± standard deviation
Age (years)	9.2 ± 5.4
	*n* (%)
Sex (males/females)	19 (48%)/21 (52%)
Diagnosis	
Pilocytic astrocytoma	16/40 (40%)
NF1+	2/16 (13%)
Ganglioglioma	6/40 (15%)
Ependymoma	11/40 (27%)
NF2+	9/11 (82%)
Subependymoma	1/40 (3%)
Diffuse midline glioma	1/40 (3%)
Anaplastic astrocytoma	3/40 (7%)
Glioblastoma	2/40 (5%)
Available genetic data	
Marker	
BRAF mutation-	7/40 (18%)
BRAF mutation+	1/40 (3%)
KIAA1549 fusion+	1/40 (3%)
H3.3K27+	1/40 (3%)

*NF1* Neurofibromatosis type 1, *NF2* Neurofibromatosis type 2

### Effect size

Cohen’s *d* effect sizes were calculated for all 120 radiomic features, once for EP *versus*. other IMSTs (EP = 11 cases, other IMSTs = 29 cases) and once for PA *versus* other IMSTs (PA = 16 cases, other IMSTs = 24 cases). The average absolute effect size was evaluated against the chance threshold to determine whether features possessed sufficient discriminability to support ML analysis.

For EP classification, the average absolute Cohen’s *d* was 0.741, exceeding the recommended threshold and indicating that many individual radiomic features discriminated EP from non-EP cases with medium-to-large effects (Supplementary Fig. S1a). Specifically, 51 of 120 features (42.5%) exhibited large effects (|d| ≥ 0.8), 23 features (19.2%) showed medium effects (0.5 ≤ |d| < 0.8), 34 features (28.3%) had small effects (0.2 ≤ |d| < 0.5), and only 12 features (10.0%) showed negligible effects (|d| < 0.2). The maximum absolute effect size was 1.808, confirming that strongly discriminative features were present in the dataset. For PA classification, the average absolute Cohen’s *d* was 0.356, falling below the 0.5 threshold (Supplementary Fig. S1b). Only 1 of 120 features (0.8%) demonstrated a large effect, while 37 features (30.8%) showed medium effects, 46 features (38.3%) showed small effects, and 36 features (30.0%) had negligible effects.

### Model development and assessment

**Fig. 4:**
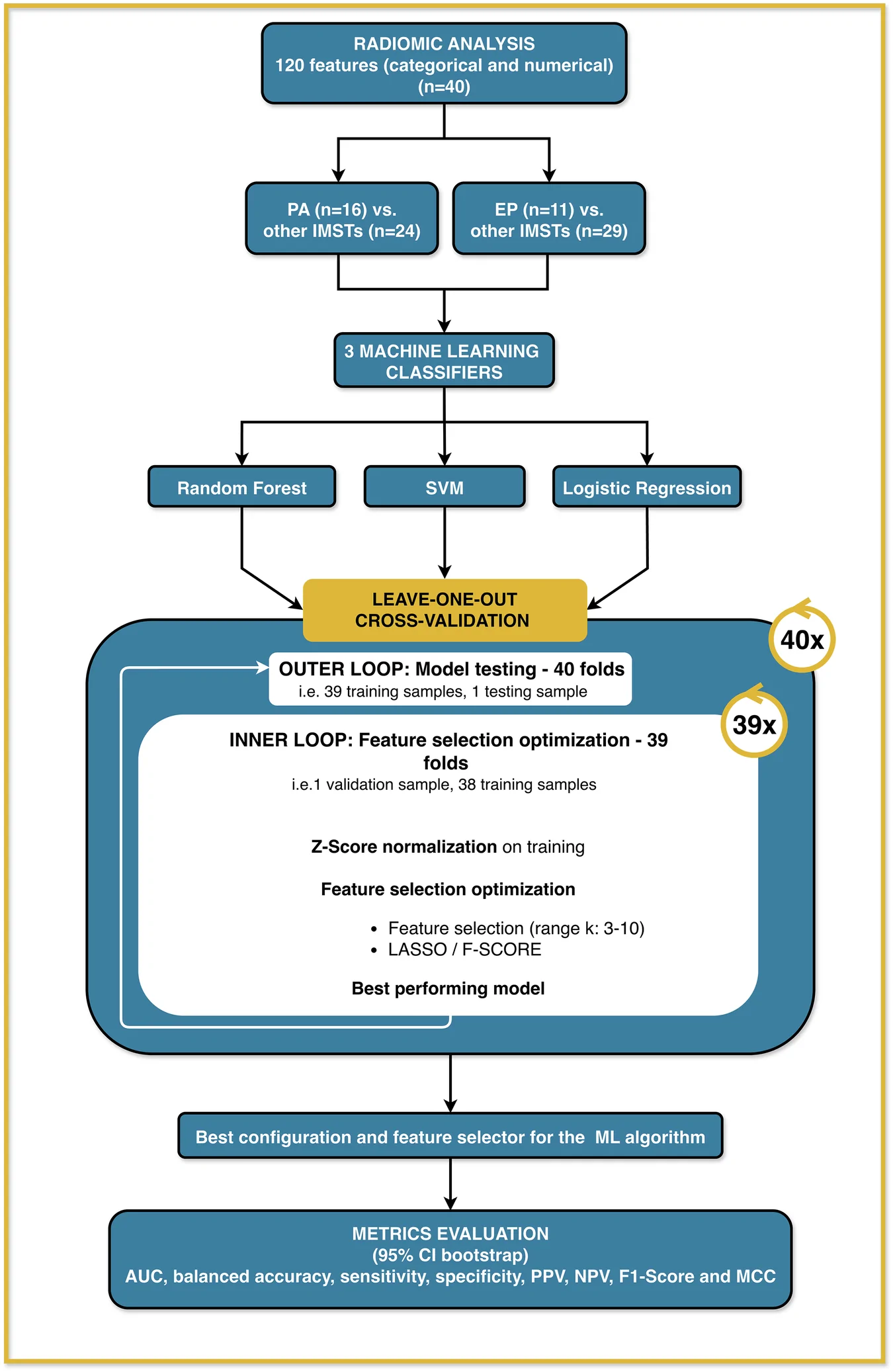
Machine learning workflow. Pipeline diagram illustrating machine learning analysis. EP, Ependymomas; IMSTs, Intramedullary spinal tumors; LASSO, Least absolute shrinkage and selection operator; MCC, Matthews correlation coefficient; ML, Machine learning; NPV, Negative predictive value; PA, Pilocytic astrocytomas; PPV, Positive predictive value; SVM, Support vector machine

The ML pipeline is illustrated in Fig. 4.

For the EP dataset, all three classifiers employing LASSO feature selector achieved a high and statistically significant classification performance (Supplementary Table S1). In particular, logistic regression achieved an AUC of 0.828 (95% CI: 0.687–0.950, *p* < 0.0001) and a balanced accuracy of 0.771 at the optimal threshold (0.253). SVM attained the highest AUC among all EP models: 0.852 (95% CI: 0.723–0.955, *p* < 0.0001), with a balanced accuracy of 0.817 at threshold 0.204. Random forest yielded the highest balanced accuracy of 0.834 at threshold 0.243, with an AUC of 0.838 (95% CI: 0.697–0.949, *p* < 0.0001).

**Fig. 5:**
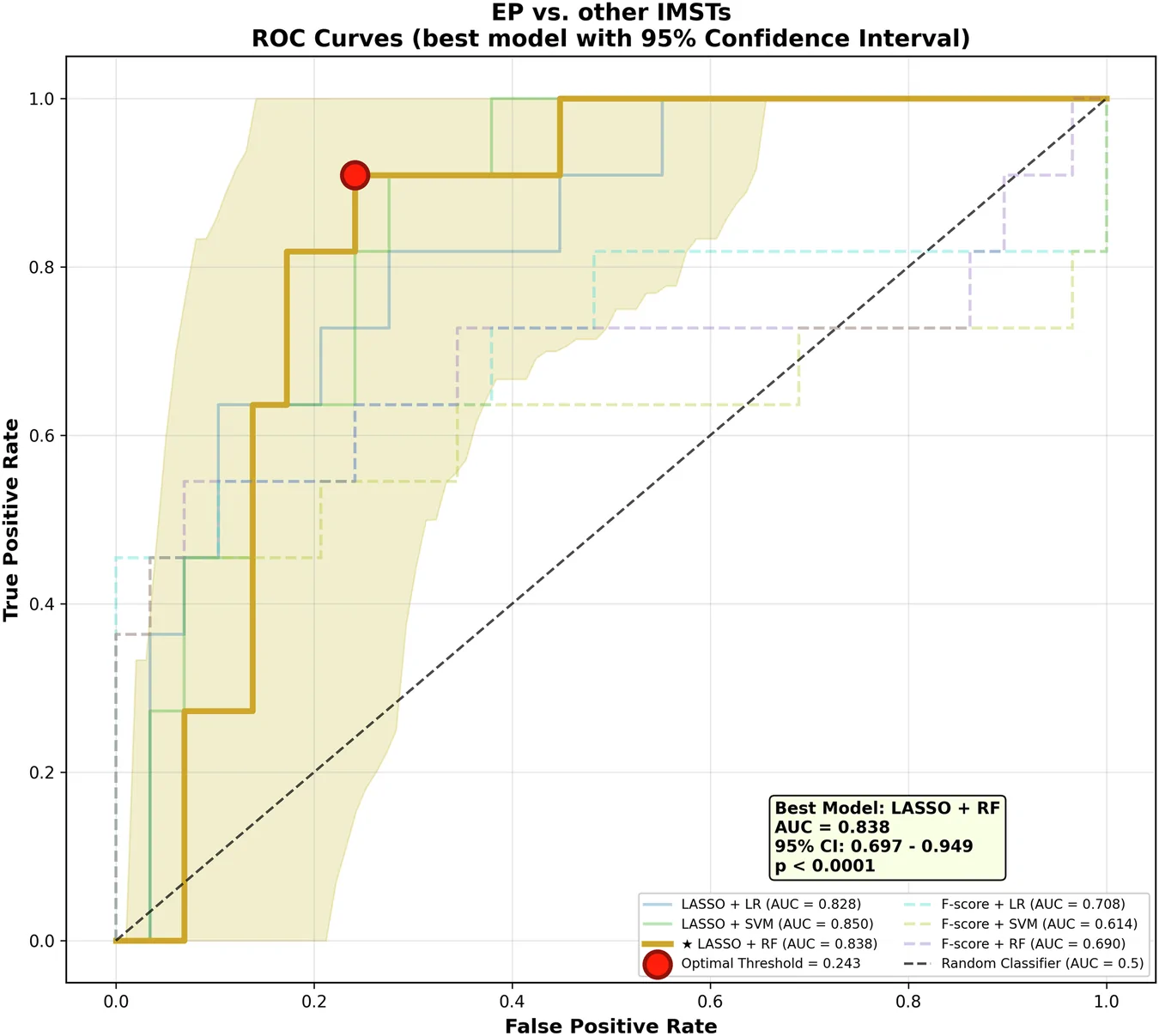
Best-performing model for ependymoma classification. ROC curves demonstrate the performance of the top-performing model in distinguishing ependymomas from other IMSTs. The random forest model with LASSO feature selection achieved the highest performance, with an AUC of 0.838. AUC, Area under the curve; EP, Ependymomas; IMSTs, Intramedullary spinal tumors; LASSO, Least absolute shrinkage and selection operator; LR, Logistic regression; RF, Random forest; ROC, Receiver operating characteristic; SVM, Support vector machine

The best overall performance, considering the balance between discriminative power (AUC) and clinical metrics, was achieved by the LASSO + random forest (Fig. 5). At its Youden-optimized threshold of 0.243, this model correctly identified 10 out of 11 EP cases (sensitivity 0.909) and 22 out of 29 non-EP cases (specificity 0.759). The confusion matrix yielded a PPV of 0.588, NPV of 0.957 (Supplementary Fig. S2). In addition, an MCC of 0.603 was calculated (Supplementary Table S1). The model used an average of 4.1 features per outer fold.

Youden optimization substantially improved performance: the balanced accuracy of LASSO + random forest increased from 0.585 at the default 0.5 threshold to 0.834 at the optimal threshold (Δ = +0.249, Table 2). Similar improvements were observed for LASSO + SVM (0.704 to 0.817, Δ = +0.113), while LASSO + logistic regression showed more modest gains (0.766 to 0.771, Δ = +0.005) (Supplementary Table S1).

**Table 2:** Performance of the Random Forest model with LASSO feature selection for classifying ependymoma *versus* other IMSTs

Metric	Th = 0.500 [95% CI]	Th = 0.243 [95% CI]
AUC	0.838* [0.697, 0.949]	-
Balanced accuracy	0.585 [0.448, 0.745]	0.834 [0.703, 0.936]
MCC	0.212 [-0.141, 0.560]	0.603 [0.352, 0.819]
Sensitivity	0.273 [0.000, 0.571]	0.909 [0.692, 1.000]
Specificity	0.897 [0.769, 1.000]	0.759 [0.600, 0.923]
F1 score	0.353 [0.000, 0.632]	0.714 [0.476, 0.882]
PPV	0.500 [0.000, 1.000]	0.588 [0.333, 0.833]
NPV	0.765 [0.622, 0.906]	0.957 [0.857, 1.000]

The table summarizes the performance metrics at the default and optimal classification thresholds for the best-performing model in distinguishing ependymoma from other IMSTs

*MCC* Matthews correlation coefficient, *Th* Threshold

* *p*-value < 0.0001

Classification performance was generally lower, although still informative, for classifiers employing F-score feature selection. Specifically, logistic regression achieved AUC 0.703 (95% CI: 0.459–0.919, *p* = 0.088), SVM achieved AUC 0.614 (95% CI: 0.360–0.875, *p* = 0.391), and random forest achieved AUC 0.691 (95% CI: 0.442–0.899, *p* = 0.112). The highest balanced accuracy among F-score models was 0.738 (random forest, threshold 0.784). Although these AUC values did not reach conventional statistical significance, the Youden-optimized thresholds produced clinically useful metrics: F-score + SVM achieved specificity of 0.966 (28/29 non-EP identified) and NPV of 0.824; F-score + logistic regression reached perfect specificity (1.000) and PPV of 1.000 at threshold 0.908, albeit at the cost of sensitivity (0.455) (Supplementary Tables S1 and S2).

Nevertheless, LASSO-based models consistently outperformed their F-score counterparts in AUC (mean LASSO AUC: 0.839 *versus* mean F-score AUC: 0.669) and balanced accuracy at the optimal threshold (mean LASSO: 0.807 *versus* mean F-score: 0.725). The Youden-optimal thresholds for the EP dataset were substantially lower than 0.5 with LASSO (range: 0.204–0.253 for LASSO; 0.596–0.908 for F-score) (Supplementary Table S1). The average number of features selected per fold was lower for LASSO (4.1–4.4) than for F-score (3.5–7.5), suggesting that LASSO’s multivariate ranking identified a more compact set of complementary features.

For the PA dataset, no model achieved clinically acceptable classification performance (Supplementary Table S3). The highest AUC was 0.584 (F-score + logistic regression; 95% CI: 0.390–0.775, *p* = 0.392), and the highest balanced accuracy was 0.677 (F-score + SVM, threshold 0.453). Notably, LASSO + SVM produced an AUC of 0.482, lower than the chance level. All AUC *p*-values for PA models exceeded 0.05, confirming that none differed significantly from random classification. For PA, neither method yielded adequate performance (mean AUC: 0.527 LASSO, 0.566 F-score). Consequently, PA classification was not pursued further. These findings supported the conclusions of our previous Cohen’s *d* effect size analysis.

### Feature stability for radiological interpretability of results

**Fig. 6:**
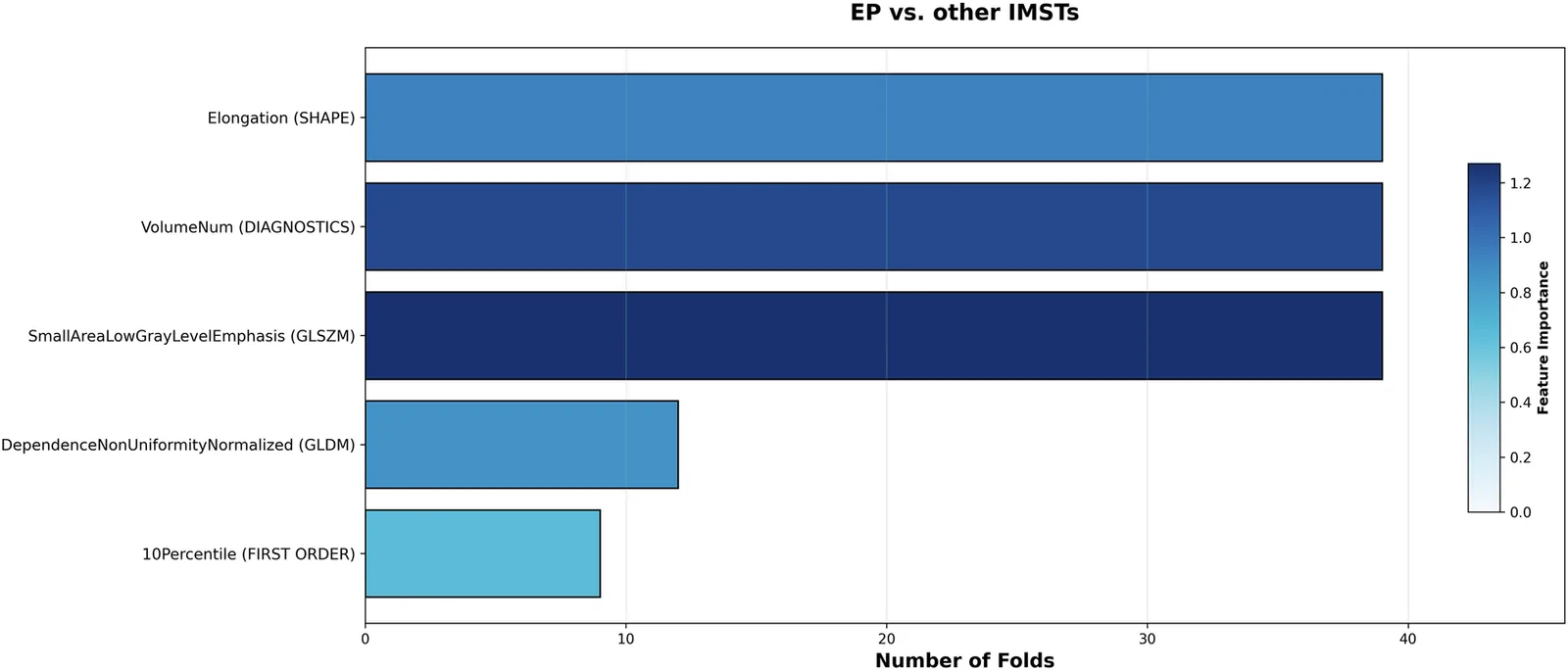
Top five most important features from the best-performing ependymoma classification model. The figure highlights the five most important features identified by the best-performing model (random forest with LASSO feature selection) for distinguishing ependymomas from other IMSTs. EP, Ependymomas; GLSZM, Gray level size zone matrix; GLDM, Gray level dependence matrix; IMSTs, Intramedullary spinal tumors; LASSO, Least absolute shrinkage and selection operator

Based on the best EP model (LASSO + random forest), the most consistently selected radiomic features were: diagnostics_Mask-interpolated_VolumeNum (selected in 39/40 outer folds), original_glszm_SmallAreaLowGrayLevelEmphasis (39/40), original_shape_Elongation (39/40), original_gldm_DependenceNonUniformityNormalized (12/40), and original_firstorder_10Percentile (9/40) (Fig. 6).

## Discussion

In our retrospective study, we analyzed three ML algorithms, each employing two feature selection methodologies to distinguish PA and EP, independently, from other pediatric IMSTs. Preliminary effect size assessment (Cohen’s *d* = 0.356) revealed radiomic features were inadequate for discriminating PAs from other IMSTs, findings corroborated by subsequent ML performance metrics. In contrast, antecedent analysis revealed 70.8% of radiomic features demonstrated medium-to-large effect sizes for EP classification (Cohen’s *d* = 0.741), suggesting discriminative utility. Random forest with the LASSO feature selection achieved superior performance (balanced accuracy 0.834; AUC 0.838), consistent with published literature on smaller cohorts [33]. Our ML pipeline is aligned with the advocated LOONCV methodology recommendations for small imaging datasets, which is employed to mitigate overfitting [34].

In our cohort, ependymomas and pilocytic astrocytomas accounted for 67% of cases, with gangliogliomas contributing 15%. Among the five most stable radiomic features distinguishing ependymomas from other IMSTs, Volume Num (MASK-INTERPOLATED class) ranked highest, suggesting that ependymomas in our cohort tended to exhibit larger tumor volumes, though extensive lesions occur across nearly all IMST histological types, limiting the diagnostic specificity of volumetric parameters alone [2]. In contrast, elongation (PyRadiomic, “SHAPE” class), ranked 3rd, may more reliably reflect tumor phenotype. While longitudinally extensive lesions can occur in intramedullary astrocytomas (multisegmental or holochord involvement in PAs) and gangliogliomas, most spinal astrocytomas typically extend craniocaudally over fewer than four vertebral body segments [35], whereas ependymomas more commonly demonstrate greater longitudinal involvement [36]. The 2nd (Small Area Low Gray Level Emphasis, GLSZM class), 4th (Dependence Non-Uniformity Normalized, GLDM class), and 5th (Minimum, FIRSTORDER class) features further suggest ependymomas contain a greater number of small T2-hypointense regions with increased textural heterogeneity, potentially reflecting hemorrhage with hemosiderin deposition and necrotic degeneration more frequently observed in ependymomas due to their highly vascular stroma [36, 37]. In contrast, astrocytomas and gangliogliomas more rarely demonstrate hemorrhagic components and typically exhibit a more homogeneous T2 signal pattern. When considered in conjunction with clinical characteristics and topography, including patient age (PAs more common in younger children, EPs in older pediatric patients), tumor location within the spinal cord (more central in EPs *versus* more eccentric in astrocytomas and gangliogliomas), and cyst location (polar in ependymomas *versus* central in astrocytomas with syrinx formation in the latter) [38, 39], these radiomic features may improve phenotype prediction. Due to our small cohort size, these findings should be interpreted cautiously and warrant validation in larger, independent cohorts.

To our knowledge, this is the first dedicated effort to classify pediatric IMSTs using radiomic features and ML. Previous research has primarily focused on tumor segmentation and grading and distinguishing IMSTs from non-neoplastic lesions. Zhuo et al developed a DL pipeline that combined automated segmentation on T2-WI, achieving 96% accuracy in differentiating tumors from demyelinating lesions, though astrocytoma-ependymoma differentiation accuracy was lower (82%) [5]. Similarly, Zhang et al achieved 93% accuracy in differentiating IMSTs from tumefactive demyelinating lesions by integrating multimodal imaging features from T2- and T1-WI [6]. Both studies were limited to cohorts comprising only ependymomas and astrocytomas. In contrast, our study sought to encompass a broader IMST spectrum and to develop a model specifically tailored for the classification of neoplastic lesions in a pediatric-specific cohort.

Radiomic-based models, including both adult and pediatric patients, have been developed to predict H3 K27 mutation status, a marker linked to poor prognosis. Li et al developed a logistic regression radiomics model using automatically segmented T2-WI to noninvasively determine H3 K27 mutation status in spinal cord diffuse midline gliomas; analyzing MRIs from 97 patients, they selected 11 radiomic features to train and test their model, achieving high accuracy and reliability. This study included a pediatric subgroup analysis, demonstrating predictive accuracies around 0.76, consistent with those observed in adults [9]. Jung et al conducted a retrospective study on a small cohort of 41 patients (ages 2–73) using conventional MRI sequences, including axial and sagittal T1-, T2-, and post-contrast T1-WI, without tumor segmentation. A random forest model analyzing clinical and imaging features identified hemorrhage as the only significant differentiating factor (*p* = 0.033). The model demonstrated moderate performance, with an AUC of 0.63. The high specificity (88.2%) suggests strong accuracy in identifying wild-type cases. However, the low sensitivity (45.8%) highlights its limited ability to detect true mutant cases, possibly due to overlapping imaging characteristics between mutant and wild-type tumors. Notably, the authors did not conduct an independent analysis on the pediatric subgroup [8].

While efforts to include younger patients in predictive models are commendable, adult-specific models remain more extensively studied and tend to exhibit higher performance [4, 7, 10, 40]. This highlights the limited applicability of such models to pediatric cohorts and underscores the scarcity of predictive tools tailored for this age group. Our findings add to the growing body of evidence supporting the value of radiomics and ML in advancing pediatric spinal oncology.

While promising for EP differentiation, the low performance across metrics in differentiating PAs suggests that these radiomic-based models are not yet suitable as standalone diagnostic tools. Recent studies emphasize integrating clinical characteristics unique to pediatric patients with IMSTs. Umebayashi et al identified significant associations between pediatric IMSTs and scoliosis, paresthesia, and paresis [41]. Additionally, imaging features of pediatric IMSTs, including tumor location, the presence of cysts, spinal cord-associated edema, and extension, have emerged as valuable biomarkers for distinguishing between different IMST types [42]. These studies emphasize the importance of integrating clinical and imaging data into ML models to enhance prediction accuracy. To improve robustness and generalizability, larger datasets, enhanced model training, and the integration of additional clinical or genetic data will be essential.

Study limitations include the retrospective design and cohort heterogeneity, particularly variations in MRI scanners and sequence parameters, which necessitated data normalization for radiomic feature extraction. However, Fisher’s exact test showed no significant association between scanner type and tumor type. Second, the single-center setting, the limited number of patients, and the small sample sizes for each tumor type limit the generalizability of findings. Third, the dataset was imbalanced, with 40% PA, 27% EP, and fewer higher-grade tumors, which may restrict the model’s applicability to rarer tumor types. Finally, a lack of histopathologic confirmation in 82% of NF2-associated EPs, despite their very strong association with phacomatoses, poses another significant limitation. In comparison, only 13% of PA lacked histologic confirmation due to their association with NF1.

Peritumoral hyperintensity on T2-WI (including fluid attenuated inversion-recovery) was deliberately excluded from the region-of-interest, and segmentation was restricted to the tumor core defined by visible lesion margins and, when present, contrast enhancement, as peritumoral signal alterations may reflect either vasogenic edema, reactive changes, and/or microscopic tumor infiltration that cannot be reliably distinguished on conventional MRI. Nonetheless, excluding the peritumoral compartment may omit diagnostically relevant information that can contribute to lesion characterization in clinical practice. Furthermore, restricting the analysis to the tumor core requires expert manual segmentation, introducing operator dependency and reducing automation. Hence, our approach should primarily be viewed as a method for investigating intrinsic tumor imaging characteristics rather than as a fully automated clinical decision-support tool. Future studies, including quantitative segmentation comparisons, should evaluate the incremental value of incorporating peritumoral abnormalities on T2-WI.

Despite these limitations, our findings contribute to the growing field of radiomics in pediatric neuro-oncology. Moving forward, future studies should focus on larger, more balanced datasets, prospective multi-institutional validation, and the integration of multimodal data. Hybrid approaches combining automated segmentation methods with radiomics-based classification models could create scalable, robust pipelines. Furthermore, incorporating clinical and genetic data could improve diagnostic accuracy and provide deeper insights into tumor biology.

Our study highlights the potential of T2-WI radiomics-based ML models for the non-invasive differentiation of ependymomas from other pediatric intramedullary spinal tumors. The low performance observed in differentiating PAs from other IMSTs emphasizes the need for further refinement and optimization. Key limitations, including the retrospective design, cohort heterogeneity, and tumor type imbalance, underscore the importance of prospective, multi-center studies with standardized imaging protocols and larger, more balanced cohorts. Additionally, incorporating a multimodal approach that integrates clinical and genetic data could further enhance diagnostic accuracy.

## Supplementary information

**Additional File 1: Fig. S1** Preliminary effect size analysis of radiomic features for ependymoma (**a**) and pilocytic astrocytoma (**b**). A substantial proportion of radiomic features demonstrated medium-to-large effect sizes for ependymoma classification (Cohen’s *d* = 0.741) (**a**). In contrast, radiomic features showed insufficient effect sizes for pilocytic astrocytoma classification (Cohen’s *d* = 0.356) (**b**). *EP* Ependymomas, *IMSTs* Intramedullary spinal tumors, *PA* Pilocytic astrocytomas **Fig. S2** Confusion matrix for the best-performing ependymoma classification model. The confusion matrix illustrates the predictions generated by the top-performing model for distinguishing ependymomas from other IMSTs. Sensitivity (Se), specificity (Sp), negative predictive value (NPV), and positive predictive value (PPV) were calculated. *EP* ependymomas, *IMSTs* Intramedullary spinal tumors. **Table S1** Performance metrics of all machine learning models and feature selection methods used for ependymoma classification. **Table S2**. Binary classification outcomes for ependymomas *versus* other IMSTs, including the number of selected features for each model. **Table S3** Performance metrics of all machine learning models and feature selection methods used for pilocytic astrocytoma classification.

## Data Availability

The datasets used and/or analyzed during the current study are available from the corresponding author on reasonable request.

## Abbreviations

CI: Confidence interval
DL: Deep learning
EP: Ependymomas
IMSTs: Intramedullary spinal tumors
LASSO: Least absolute shrinkage and selection operator
LOOCV: Leave-one-out cross-validation
MCC: Matthews correlation coefficient
ML: Machine learning
NPV: Negative predictive value
PA: Pilocytic astrocytomas
PPV: Positive predictive value
SVM: Support vector machine
T1-WI: T1-weighted images
T2-WI: T2-weighted images

## References

[1] Ostrom QT, Price M, Neff C et al (2023) CBTRUS statistical report: primary brain and other central nervous system tumors diagnosed in the United States in 2016–2020. Neuro Oncol 25:iv1–iv99. 10.1093/neuonc/noad149 37793125 PMC10550277

[2] Shih RY, Koeller KK (2020) Intramedullary masses of the spinal cord: radiologic-pathologic correlation. Radiographics 40:1125–1145. 10.1148/rg.2020190196 32530746

[3] van Timmeren JE, Cester D, Tanadini-Lang S et al (2020) Radiomics in medical imaging—“how-to” guide and critical reflection. Insights Imaging 11:91. 10.1186/s13244-020-00887-2 32785796 PMC7423816

[4] Lemay A, Gros C, Zhuo Z et al (2021) Automatic multiclass intramedullary spinal cord tumor segmentation on MRI with deep learning. Neuroimage Clin 31:102766. 10.1016/j.nicl.2021.102766 34352654 PMC8350366

[5] Zhuo Z, Zhang J, Duan Y et al (2022) Automated classification of intramedullary spinal cord tumors and inflammatory demyelinating lesions using deep learning. Radiol Artif Intell 4:e210292. 10.1148/ryai.210292 36523644 PMC9745442

[6] Zhang Z, Li N, Qian Y, Cheng H (2024) Establishment of an MRI-based radiomics model for distinguishing between intramedullary spinal cord tumor and tumefactive demyelinating lesion. BMC Med Imaging 24:317. 10.1186/s12880-024-01499-8 39574000 PMC11583559

[7] Ma C, Wang L, Song D et al (2023) Multimodal-based machine learning strategy for accurate and non-invasive prediction of intramedullary glioma grade and mutation status of molecular markers: a retrospective study. BMC Med 21:198. 10.1186/s12916-023-02898-4 37248527 PMC10228074

[8] Jung JS, Choi YS, Ahn SS et al (2019) Differentiation between spinal cord diffuse midline glioma with histone H3 K27M mutation and wild type: comparative magnetic resonance imaging. Neuroradiology 61:313–322. 10.1007/s00234-019-02154-8 30662997

[9] Li J, Wang Y, Weng J et al (2023) Automated determination of the H3 K27-altered status in spinal cord diffuse midline glioma by radiomics based on T2-weighted MR images. AJNR Am J Neuroradiol 44:1464–1470. 10.3174/ajnr.A8056 38081676 PMC10714849

[10] Sun T, Wang Y, Liu X et al (2023) Deep learning based on preoperative magnetic resonance (MR) images improves the predictive power of survival models in primary spinal cord astrocytomas. Neuro Oncol 25:1157–1165. 10.1093/neuonc/noac280 36562243 PMC10237430

[11] Zwanenburg A, Vallières M, Abdalah MA et al (2020) The image biomarker standardization initiative: standardized quantitative radiomics for high-throughput image-based phenotyping. Radiology 295:328–338. 10.1148/radiol.2020191145 32154773 PMC7193906

[12] Wichtmann BD, Harder FN, Weiss K et al (2023) Influence of image processing on radiomic features from magnetic resonance imaging. Invest Radiol 58:199–208. 10.1097/RLI.0000000000000921 36070524

[13] Koçak B, Yüzkan S, Mutlu S et al (2024) Influence of image preprocessing on the segmentation-based reproducibility of radiomic features: *in vivo* experiments on discretization and resampling parameters. Diagn Interv Radiol 30:152–162. 10.4274/dir.2023.232543 38073244 PMC11095065

[14] van Griethuysen JJM, Fedorov A, Parmar C et al (2017) Computational radiomics system to decode the radiographic phenotype. Cancer Res 77:e104–e107. 10.1158/0008-5472.CAN-17-0339 29092951 PMC5672828

[15] Rajput D, Wang W-J, Chen C-C (2023) Evaluation of a decided sample size in machine learning applications. BMC Bioinform 24:48. 10.1186/s12859-023-05156-9 PMC9926644 36788550

[16] Kleinbaum DG (1994) Introduction to Logistic Regression. In: Logistic Regression. Statistics in the Health sciences. Springer, New York.

[17] Breiman L (2001) Random forests. Mach Learn 45:5–32. 10.1023/A:1010933404324

[18] Shmilovici A (2005) Support vector machines. In: Maimon O, Rokach L (eds) Data mining and knowledge discovery handbook. Springer, Boston. 10.1007/0-387-25465-X_12

[19] Buitinck L, Louppe G, Blondel M et al (2013) API design for machine learning software: experiences from the scikit-learn project. Preprint at 10.48550/arXiv.1309.0238

[20] Van Rossum G, Drake FL (2009) Python 3 reference manual. CreateSpace, Scotts Valley

[21] Peng C-YJ, Lee KL, Ingersoll GM (2002) An introduction to logistic regression analysis and reporting. J Educ Res 96:3–14. 10.1080/00220670209598786

[22] Chauhan VK, Dahiya K, Sharma A (2019) Problem formulations and solvers in linear SVM: a review. Artif Intell Rev 52:803–855. 10.1007/s10462-018-9614-6

[23] Vabalas A, Gowen E, Poliakoff E, Casson AJ (2019) Machine learning algorithm validation with a limited sample size. PLoS One 14:e0224365. 10.1371/journal.pone.0224365 31697686 PMC6837442

[24] Chang C-C, Liu T-C, Lu C-J et al (2023) Machine learning strategy for identifying altered gut microbiomes for diagnostic screening in myasthenia gravis. Front Microbiol 14:1227300. 10.3389/fmicb.2023.1227300 37829445 PMC10565662

[25] Bisdas S, Shen H, Thust S et al (2018) Texture analysis- and support vector machine-assisted diffusional kurtosis imaging may allow *in vivo* gliomas grading and IDH-mutation status prediction: a preliminary study. Sci Rep 8:6108. 10.1038/s41598-018-24438-4 29666413 PMC5904150

[26] Raschka S (2020) Model evaluation, model selection, and algorithm selection in machine learning. 10.48550/arXiv.1811.12808

[27] Thölke P, Mantilla-Ramos Y-J, Abdelhedi H et al (2023) Class imbalance should not throw you off balance: choosing the right classifiers and performance metrics for brain decoding with imbalanced data. Neuroimage 277:120253. 10.1016/j.neuroimage.2023.120253 37385392

[28] Chicco D, Jurman G (2023) The Matthews correlation coefficient (MCC) should replace the ROC AUC as the standard metric for assessing binary classification. BioData Min 16:4. 10.1186/s13040-023-00322-4 36800973 PMC9938573

[29] Foody GM (2023) Challenges in the real-world use of classification accuracy metrics: from recall and precision to the Matthews correlation coefficient. PLoS One 18:e0291908. 10.1371/journal.pone.0291908 37792898 PMC10550141

[30] Lobo JM, Jiménez-Valverde A, Real R (2008) AUC: a misleading measure of the performance of predictive distribution models. Glob Ecol Biogeogr 17:145–151. 10.1111/j.1466-8238.2007.00358.x

[31] Fluss R, Faraggi D, Reiser B (2005) Estimation of the Youden index and its associated cutoff point. Biom J 47:458–472. 10.1002/bimj.200410135 16161804

[32] Lydersen S, Pradhan V, Senchaudhuri P, Laake P (2007) Choice of test for association in small sample unordered r x c tables. Stat Med 26:4328–4343. 10.1002/sim.2839 17311220

[33] Katzmann A, Mühlberg A, Suehling M et al (2020) Deep random forests for small sample size prediction with medical imaging data. In: Proceedings of the 2020 IEEE 17th international symposium on biomedical imaging (ISBI). IEEE, Iowa City, pp 1543–1547

[34] Bradshaw TJ, Huemann Z, Hu J, Rahmim A (2023) A guide to cross-validation for artificial intelligence in medical imaging. Radiol Artif Intell 5:e220232. 10.1148/ryai.220232 37529208 PMC10388213

[35] Deora H, Sumitra S, Nandeesh BN et al (2019) Spinal intramedullary ganglioglioma in children: an unusual location of a common pediatric tumor. Pediatr Neurosurg 54:245–252. 10.1159/000500427 31212295

[36] Moore KR, Linscott LL (2024) Spinal ependymoma. In: Diagnostic imaging: pediatric neuroradiology, 4th edn. Elsevier, Salt Lake City

[37] Kobayashi K, Ando K, Kato F et al (2018) MRI characteristics of spinal ependymoma in WHO grade II: a review of 59 cases. Spine (Phila Pa 1976) 43:E525–E530. 10.1097/BRS.0000000000002496 29189641

[38] Kim DH, Kim J-H, Choi SH et al (2014) Differentiation between intramedullary spinal ependymoma and astrocytoma: comparative MRI analysis. Clin Radiol 69:29–35. 10.1016/j.crad.2013.07.017 24034546

[39] She D-J, Lu Y-P, Xiong J et al (2019) MR imaging features of spinal pilocytic astrocytoma. BMC Med Imaging 19:5. 10.1186/s12880-018-0296-y 30642288 PMC6332544

[40] Chianca V, Cuocolo R, Gitto S et al (2021) Radiomic machine learning classifiers in spine bone tumors: a multi-software, multi-scanner study. Eur J Radiol. 10.1016/j.ejrad.2021.109586 33610852

[41] Umebayashi D, Naito K, Kurokawa R et al (2024) Epidemiology and comparative analysis of outcomes of intramedullary spinal cord tumor between pediatric and adult patients. Spine (Phila Pa 1976) 49:107–115. 10.1097/BRS.0000000000004775 37466205

[42] Cerron-Vela CR, Gonçalves FG, Tierradentro-García LO et al (2024) A comprehensive evaluation of imaging features in pediatric spinal gliomas and their value in predicting tumor grade and histology. Neuroradiology 66:1311–1324. 10.1007/s00234-024-03395-y 38902483 PMC11246280

