# Supplementary material for: Radiomics and machine learning for differentiating pediatric intramedullary spinal tumors: a pilot study
Source: Eur Radiol Exp. 2026 Sep 21;10:140. doi: 10.1186/s41747-026-00810-2 (PMC13594037; doi:10.1186/s41747-026-00810-2)
Supplement: Supplementary file 1 [file 41747_2026_810_MOESM1_ESM.pdf]

Radiomics and machine learning for differentiating pediatric intramedullary spinal tumors: a pilot study

ELECTRONIC SUPPLEMENTARY MATERIAL

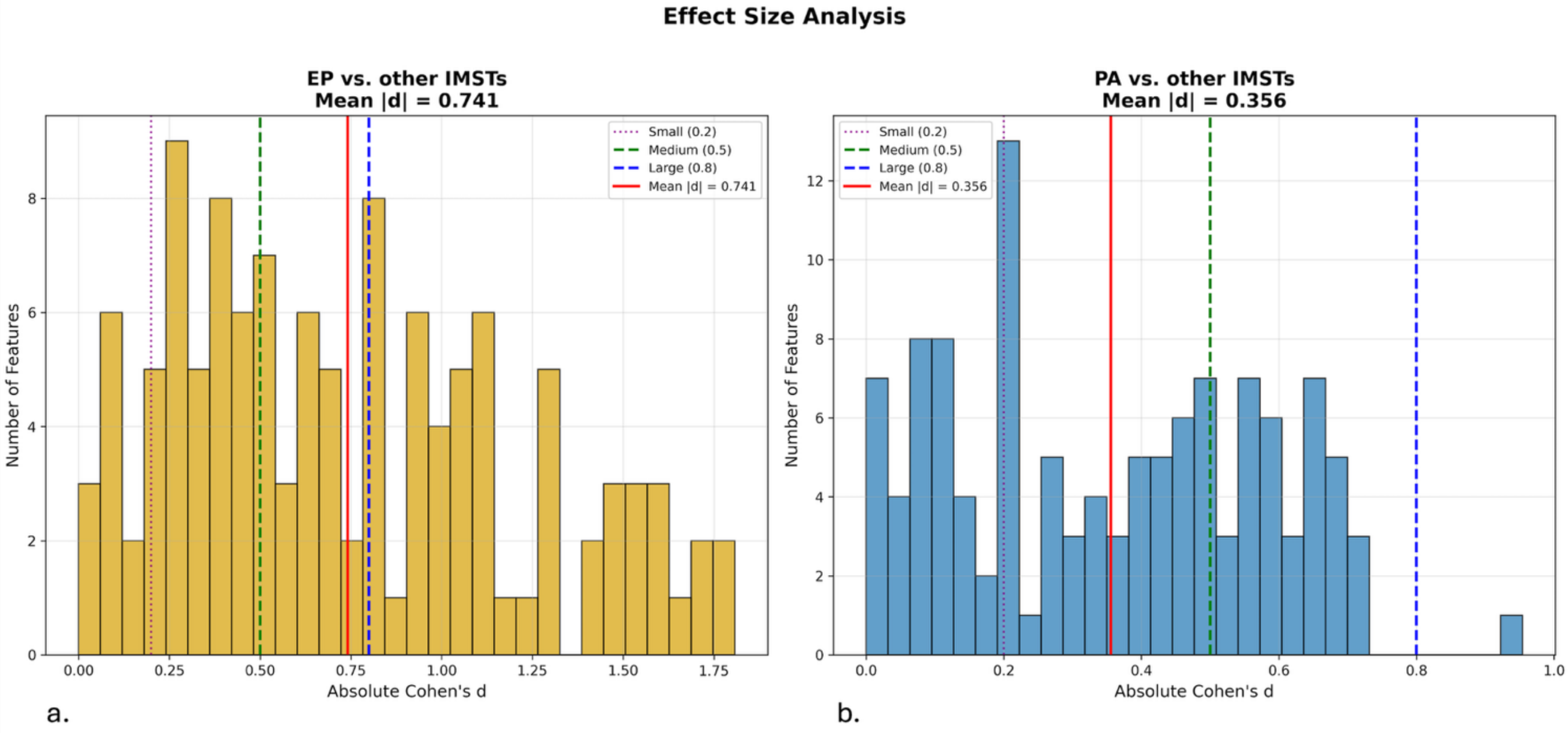

Fig. S1 Preliminary effect size analysis of radiomic features for ependymoma (**a**) and pilocytic astrocytoma (**b**). A substantial proportion of radiomic features demonstrated medium-to-large effect sizes for ependymoma classification (Cohen's d = 0.741) (**a**). In contrast, radiomic features showed insufficient effect sizes for pilocytic astrocytoma classification (Cohen's d = 0.356) (**b**). *EP* Ependymomas, *IMSTs* Intramedullary spinal tumors, *PA* Pilocytic astrocytomas

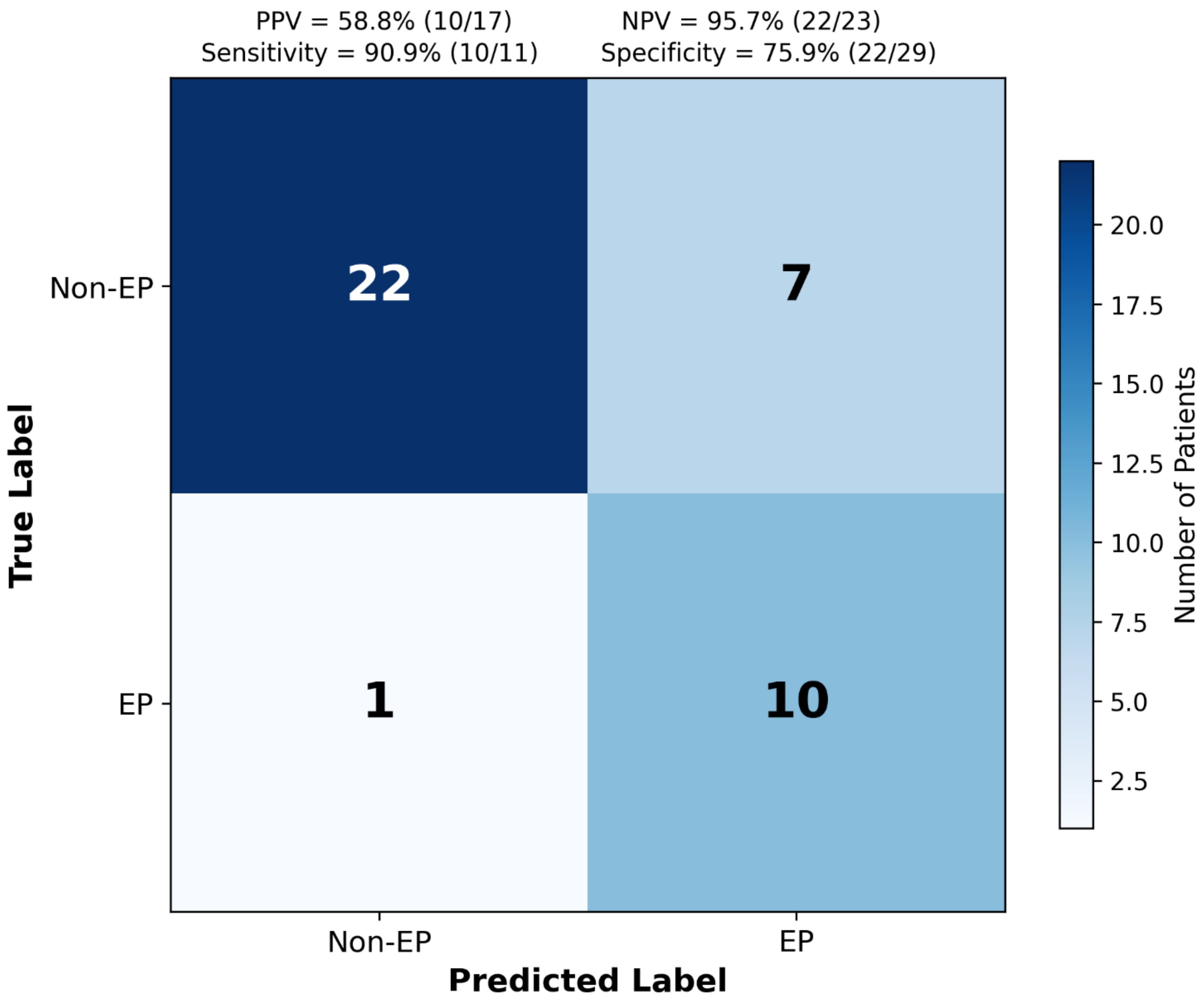

Fig. S2 Confusion matrix for the best-performing ependymoma classification model. The confusion matrix illustrates the predictions generated by the top-performing model for distinguishing ependymomas from other IMSTs. Sensitivity (Se), specificity (Sp), negative predictive value (NPV), and positive predictive value (PPV) were calculated. *EP* ependymomas, *IMSTs* Intramedullary spinal tumors.

Table S1 Performance metrics of all machine learning models and feature selection methods used for ependymoma classification

EP *versus* other IMSTs	LASSO + Logistic Regression		LASSO + SVM (Linear)		# LASSO + Random Forest		F-score + Logistic Regression		F-Score + SVM (Linear)		F-score + Random Forest	
Metric	Th = 0.500 [95% CI]	Th = 0.253 [95% CI]	Th = 0.500 [95% CI]	Th = 0.204 [95% CI]	Th = 0.500 [95% CI]	Th = 0.243 [95% CI]	Th = 0.500 [95% CI]	Th = 0.908 [95% CI]	Th = 0.500 [95% CI]	Th = 0.596 [95% CI]	Th = 0.500 [95% CI]	Th = 0.784 [95% CI]
AUC	0.828* [0.687, 0.950]	-	0.852* [0.723, 0.955]	-	0.838* [0.697, 0.949]	-	0.703 [0.459, 0.919]	-	0.614 [0.360, 0.875]	-	0.691 [0.442, 0.899]	-
Balanced Accuracy	0.766 [0.602, 0.913]	0.771 [0.619, 0.889]	0.704 [0.547, 0.857]	0.817 [0.687, 0.924]	0.585 [0.448, 0.745]	0.834 [0.703, 0.936]	0.704 [0.541, 0.863]	0.727 [0.577, 0.885]	0.710 [0.556, 0.866]	0.710 [0.556, 0.866]	0.704 [0.547, 0.867]	0.738 [0.582, 0.891]
MCC	0.550 [0.218, 0.827]	0.490 [0.200, 0.724]	0.420 [0.099, 0.709]	0.568 [0.318, 0.792]	0.212 [-0.141, 0.560]	0.603 [0.352, 0.819]	0.420 [0.085, 0.734]	0.614 [0.348, 0.840]	0.525 [0.175, 0.804]	0.525 [0.175, 0.804]	0.420 [0.104, 0.741]	0.532 [0.190, 0.812]
Sensitivity	0.636 [0.333, 0.909]	0.818 [0.545, 1.000]	0.545 [0.250, 0.818]	0.909 [0.692, 1.000]	0.273 [0.000, 0.571]	0.909 [0.692, 1.000]	0.545 [0.250, 0.846]	0.455 [0.154, 0.769]	0.455 [0.154, 0.769]	0.455 [0.154, 0.769]	0.545 [0.250, 0.846]	0.545 [0.250, 0.846]
Specificity	0.897 [0.769, 1.000]	0.724 [0.545, 0.889]	0.862 [0.724, 0.969]	0.724 [0.545, 0.889]	0.897 [0.769, 1.000]	0.759 [0.600, 0.923]	0.862 [0.733, 0.969]	1000 [1.000, 1.000]	0.966 [0.889, 1.000]	0.966 [0.889, 1.000]	0.862 [0.733, 0.968]	0.931 [0.821, 1.000]
F1 Score	0.667 [0.375, 0.870]	0.643 [0.381, 0.818]	0.571 [0.286, 0.783]	0.690 [0.451, 0.857]	0.353 [0.000, 0.632]	0.714 [0.476, 0.882]	0.571 [0.285, 0.800]	0.625 [0.266, 00870]	0.588 [0.250, 0.833]	0.588 [0.250, 0.833]	0.571 [0.286, 0.800]	0.632 [0.333, 0.849]
PPV	0.700 [0.375, 1.000]	0.529 [0.278, 0.769]	0.600 [0.286, 0.889]	0.556 [0.316, 0.786]	0.500 [0.000, 1.000]	0.588 [0.333, 0.833]	0.600 [0.286, 0.900]	1000 [1.000, 1.000]	0.833 [0.400, 1.000]	0.833 [0.400, 1.000]	0.600 [0.272, 0.900]	0.750 [0.400, 1.000]
NPV	0.867 [0.750, 0.970]	0.913 [0.792, 1.000]	0.833 [0.710, 0.958]	0.955 [0.850, 1.000]	0.765 [0.622, 0.906]	0.957 [0.857, 1.000]	0.833 [0.700, 0.962]	0.829 [0.710, 0.944]	0.824 [0.697, 0.943]	0.824 [0.697, 0.943]	0.833 [0.700, 0.963]	0.844 [0.720, 0.963]

*p-value<0.0001, #=best performing model, MCC=Matthews Correlation Coefficient, Th = threshold

Table S2. Binary classification outcomes for ependymomas versus other IMSTs, including the number of selected features for each model

	LASSO + logistic regression	LASSO + SVM (linear)	# LASSO + random forest	F-score + logistic regression	F-score + SVM (linear)	F-score + random forest
True positives	9	10	10	5	5	6
True negatives	21	21	22	29	28	27
False positives	8	8	7	0	1	2
False negatives	2	1	1	6	6	5
Average k features	4.4	4.0	4.1	3.5	3.8	7.5

# = Best model. *LASSO* Least absolute shrinkage and selection operator, *SVM* Support vector machine.

Table S3 Performance metrics of all machine learning models and feature selection methods used for pilocytic astrocytoma classification

PA *versus* other IMSTs	LASSO + logistic regression		LASSO + SVM (linear)		LASSO + random forest		F-score + logistic regression		F-score + SVM (linear)		F-score + random forest	
Metric	Th = 0.500 [95% CI]	Th = 0.691 [95% CI]	Th = 0.500 [95% CI]	Th = 0.590 [95% CI]	Th = 0.500 [95% CI]	Th = 0.725 [95% CI]	Th = 0.500 [95% CI]	Th = 0.654 [95% CI]	Th = 0.500 [95% CI]	Th = 0.453 [95% CI]	Th = 0.500 [95% CI]	Th = 0.502 [95% CI]
AUC	0.517 [0.331, 0.701]	-	0.482 [0.271, 0.701]	-	0.582 [0.390, 0.767]	-	0.584 [0.390, 0.775]	-	0.575 [0.370, 0.775]	-	0.539 [0.336, 0.741]	-
Balanced accuracy	0.510 [0.348, 0.661]	0.562 [0.407, 0.715]	0.615 [0.452, 0.767]	0.646 [0.495, 0.791]	0.573 10.417, 0.735]	0.635 [0.522, 0.766]	0.573 [0.412, 0.730]	0.656 [0.508, 0.799]	0.562 [0.434, 0.693]	0.677 [0.527, 0.821]	0.604 [0.464, 0.755]	0.604 [0.464, 0.755]
MCC	0.021 [-0.298, 0.313]	0.134 [-0.200, 0.435]	0.227 [-0.089, 0.527]	0.305 [-0.011, 0.600]	0.150 [-0.171, 0.471]	0.372 [0.076, 0.627]	0.143 [-0.173, 0.455]	0.354 [0.019, 0.630]	0.161 [-0.175, 0.454]	0.364 [0.061, 0.646]	0.236 [-0.087, 0.549]	0.236 [-0.087, 0.549]
Sensitivity	0.562 [0.308, 0.812]	0.375 [0.133, 0.625]	0.562 [0.308, 0.812]	0.500 [0.250, 0.737]	0.438 [0.211, 0.692]	0.312 [0.091, 0.562]	0.562 [0.308, 0.812]	0.438 [0.188, 0.692]	0.250 [0.059, 0.500]	0.562 [0.308, 0.812]	0.375 [0.143, 0.632]	0.375 [0.143, 0.632]
Specificity	0.458 [0.263, 0.667]	0.750 [0.565, 0.913]	0.667 [0.480, 0.850]	0.792 [0.600, 0.950]	0.708 [0.520, 0.882]	0.958 [0.857, 1.000]	0.583 [0.368, 0.783]	0.875 [0.720, 1.000]	0.875 [0.731, 1.000]	0.792 [0.609, 0.947]	0.833 [0.667, 0.962]	0.833 [0.667,0.962]
F1 Score	0.474 [0.242, 0.652]	0.429 [0.167, 0.640]	0.545 [0.296, 0.727]	0.552 [0.296, 0.743]	0.467 [0.231, 0.686]	0.455 [0.154, 0.696]	0.514 [0.267,0.698]	0.538 [0.261, 0.741]	0.348 [0.095, 0.584]	0.600 [0.348, 0.788]	0.462 [0.190, 0.690]	0.462 [0.190, 0.690]
PPV	0.409 [0.200, 0.625]	0.500 [0.200, 0.778]	0.529 [0.286, 0.765]	0.615 [0.333, 0.875]	0.500 [0.235, 0.769]	0.833 [0.498, 1.000]	0.474 [0.250, 0.714]	0.700 [0.400, 1.000]	0.571 [0.167, 1.000]	0.643 [0.385, 0.900]	0.600 [0.285, 0.909]	0.600 [0.285, 0.909]
NPV	0.611 [0.375, 0.824]	0.643 [0.461, 0.818]	0.696 [0.481, 0.870]	0.704 [0.519, 0.864]	0.654 [0.462, 0.828]	0.676 [0.512, 0.829]	0.667 [0.450, 0.857]	0.700 [0.533, 0.857]	0.636 [0.457, 0.788]	0.731 [0.542, 0.889]	0.667 [0.500, 0.833]	0.667 [0.500, 0.833]

AUC Area under the receiver operating characteristic curve, *CI* Confidence interval, *IMSTs* Intramedullary spinal tumors, *MCC* Matthews correlation coefficient, *LASSO* Least absolute shrinkage and selection operator, *NPV* Negative predictive value, *PPV* Positive predictive value, *SVM* Support vector machine, *Th* Threshold.
